# Hippocampal volume, social interactions, and the expression of the normal repertoire of resident–intruder behavior

**DOI:** 10.1002/brb3.775

**Published:** 2017-08-07

**Authors:** Eszter Kalman, Kevin A. Keay

**Affiliations:** ^1^ School of Medical Sciences (Anatomy & Histology) The University of Sydney Sydney NSW Australia

**Keywords:** corticosterone, dominance behavior, hippocampus morphology, housing, nerve injury, rat, social buffering

## Abstract

**Introduction:**

Reduced hippocampal volumes are reported in individuals with disrupted emotional coping behaviors in both human clinical conditions and in experimental animal models of these populations. In a number of experimental animal models, it has been shown that social interactions can promote resilience and buffer the negative neural consequences of stimuli that disrupt effective coping.

**Methods:**

Hippocampal and dentate gyrus volumes were calculated in 54 male Sprague Dawley rats; (1) single housed (*n* = 12), (2) single housed and exposed to daily 6‐min social interactions testing in a resident–intruder paradigm (*n* = 11); (3) group housed (*n* = 12); (4) single housed and sham injured (*n* = 12); (5) single housed, sham injured, and social interactions tested (*n* = 7).

**Results:**

We present data which shows that even a brief daily exposure to a conspecific in resident–intruder social interactions test is sufficient to prevent the reduction in hippocampal volume triggered by single housing.

**Conclusion:**

When considered with previously published data, these findings suggest that the expression of the full repertoire of social, nonsocial, dominance, and submissive behaviors in response to the physical presence of an *intruder* in the home cage plays a significant role in this maintenance of hippocampal volume.

## 1 INTRODUCTION

Reduced hippocampal volumes are reported in patients with posttraumatic stress disorder or major depressive disorder in whom emotional coping behaviors are disrupted (Gilbertson et al., 2002; O'Doherty, Chitty, Saddiqui, Bennett, & Lagopoulos, 2015; Saylam, Üçerler, Kitiş, Ozand, & Gönül, 2006; Sheline, Gado, & Kraemer, 2003; Sheline, Sanghavi, Mintun, & Gado, 1999). In a preclinical animal model of nerve injury, similar findings have been reported in injured rats with comorbid alterations in emotional coping behaviors, an effect that is not seen in nerve‐injured rats whose emotional coping behaviors are unchanged (Kalman & Keay, 2014). Specifically, our previously published data showed that following sciatic nerve injury, in a subgroup of rats, the repertoire of behaviors usually displayed by a rat in response to the presence of an intruder into the territory of their home cage is substantially changed. This effect on resident–intruder social interactions is characterized by significantly reduced dominance behaviors and more frequent approach–avoid like behaviors (Kalman & Keay, 2014; Monassi, Bandler, & Keay, 2003). Rats with reduced dominance behaviors had significantly smaller hippocampal volumes compared to both nerve injured rats whose resident–intruder social interactions were unchanged, and sham‐injured rats with normal levels of dominance behavior (Kalman & Keay, 2014).

The coincident hippocampal volume changes and reduction of dominance behavior in injured rats raise the question of whether it is the expression of the full repertoire of resident–intruder social behaviors that in some way *protects* the hippocampus from such *shrinkage*. In the *visible burrow system* paradigm (Blanchard et al., 1995), dominant rats are readily identified by their behaviors and their levels of dominance are related to increased hippocampal neurogenesis. Dominant rats have more cells in the dentate gyrus of the hippocampus than subordinate rats, an increase that is related primarily to the survival of new neurons rather than increased levels of proliferation (Kozorovitskiy & Gould, 2004). It is often inferred, although not experimentally demonstrated, that changes in hippocampal volume are a consequence of either altered neurogenesis or the altered survival of newly generated neurons. It is possible therefore that the relationship between the expression of dominance behaviors and enhanced survival of hippocampal neurons relates to our observation that rats who do not show the normal repertoire of resident–intruder social behaviors have smaller hippocampal volumes.

In the resident–intruder test, *resident* rats are singly housed to enable the establishment of a territory in the home cage. It is important to note that each rat is in visual, auditory, and olfactory contact with other identically housed individuals, however, they do not have physical contact. Social interactions with a conspecific *intruder*, introduced into the home cage are measured daily (Monassi et al., 2003). In this study, we investigated whether the expression of the normal repertoire of resident–intruder social behaviors maintains hippocampal volume. Specifically, we measured hippocampal volumes in: (1) rats able to express the normal repertoire of social behaviors in the resident–intruder test; (2) rats similarly housed with no opportunity to express the normal repertoire of social behaviors; and (3) rats housed in a social group under standard laboratory housing conditions.

In an earlier study looking at the impact of nerve injury on hippocampal volume, we reported that nerve‐injured, sham‐injured, and uninjured rats each of which showed the normal repertoire of social behaviors in the resident–intruder test had identical hippocampal volumes. We followed up these observations in this study and compared sham‐injured rats that had undergone social interactions testing, with sham‐injured rats with no opportunity to express the normal repertoire of social behaviors in the resident–intruder test. We hypothesized a reduction in hippocampal volume in the group of rats with no opportunity to express the normal levels of dominance behavior.

## 2 METHODS

Fifty‐four male Sprague Dawley rats were used in this experiment. The University of Sydney, Animal Care and Ethics Committee approved all procedures (#3920, #4852, and #776). The rats were randomly allocated into five groups: (1) single housed (*n* = 12); (2) single housed and social interactions tested (*n* = 11); (3) group housed (*n* = 12); (4) single housed and sham injured (*n* = 12); (5) single housed, sham injured, and social interactions tested (*n* = 7).

Rats were single housed in a clear Perspex cage (40 × 36 × 24 cm) to which they were habituated (14 days). The group housed rats lived in polycarbonate cages with a wire lid (53 × 37 × 25 cm) in groups of 4–6. Food and water were available ad libitum. All rats were housed and tested under a reversed dark–light cycle (12 h:12 h). Resident–intruder social interactions testing was conducted on groups 2 and 5 as described earlier. The resident–intruder testing procedure was modified from that described by Koolhaas et al. (2013) and is described in detail by Monassi et al. (2003). In brief, an age, weight, and sex‐matched *intruder* was introduced into the home cage of each singly housed rat. The interactions of the two rats were recorded for 6 min using an infrared camera (DCRA‐C155; Sony). The behaviors of each resident rat were scored from the video record and classified into one of the four mutually exclusive categories. *Dominance behavior*: standing on top of the supine intruder, biting, chasing, aggressive grooming, boxing, and sideway lateral pushing. *Social behavior*: sniffing and exploration of the intruder specifically focused around the anogenital region. *Nonsocial*: cage exploration and self‐grooming. *Submissive*: defensive alerting, fleeing behavior, and supine postures.

Rats were behaviorally tested for 11 days (i.e., 5 test days, a *rest* day, and then 6 further test days). The rats receiving a sham injury were tested for 5 days prior to the injury, testing was not conducted on the day of surgery, then testing resumed for 6 days after the surgery. In the behavioral test, the same *intruder* rat never met a *resident* rat more than twice and never on consecutive days.

**Figure 1:**
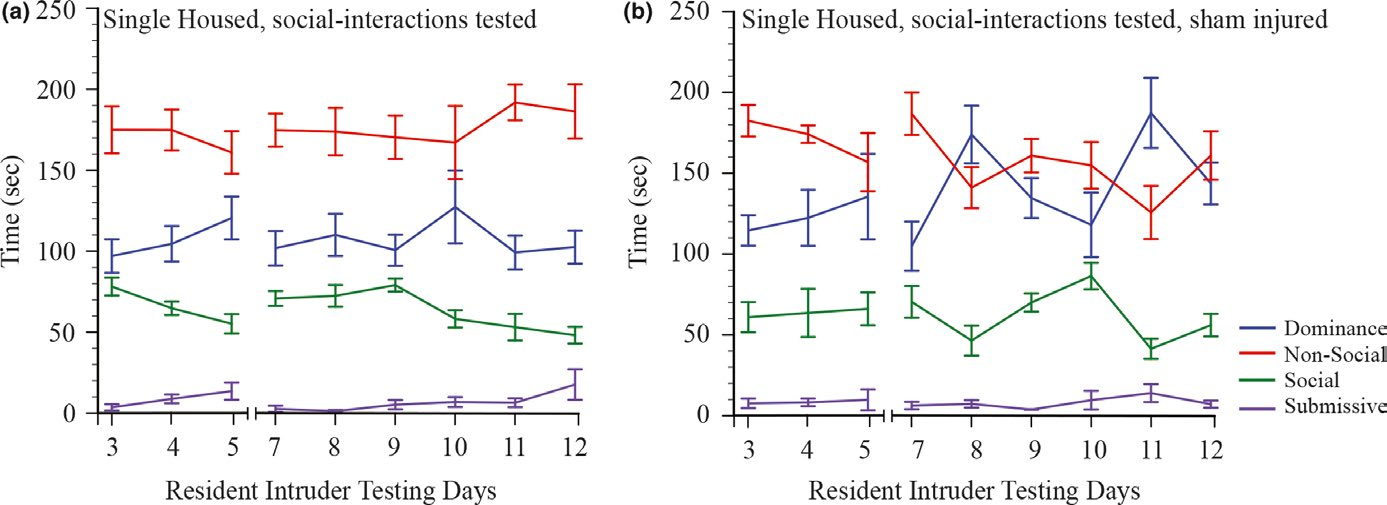
Mean durations (±SEM) of dominance, nonsocial, social, and submissive behaviors expressed by (a) single‐housed and social interactions tested rats and (b) single‐housed, social interactions tested, and sham‐injured rats on days 3–12 of resident–intruder social interactions testing

Rats in groups 4 and 5 were anesthetized with isoflurane (2%–3% adjusted) to perform the sham surgery. An incision was made in the skin overlying the right thigh muscles. The muscles were parted gently by blunt dissected and the sciatic nerve revealed at its trifurcation. The skin was then sutured and the rat was allowed to recover under close observation. The period of anesthesia and surgical procedures lasted no longer than 20 min (for further detail, see Kalman & Keay, 2014). On completion of resident–intruder testing, the brain of each rat was removed following transcardial perfusion with saline followed by paraformaldehyde (4%). A one in five series of coronal 40 μmol/L sections were cut on a cryostat, mounted on slides, and Nissl stained. A photomicrograph (40× magnification) was taken of each section and image analysis software was used to calculate the surface area of the hippocampus and dentate gyrus on every section (Schneider, Rasband & Eliceiri, 2012). Hippocampal and dentate gyrus boundaries were drawn onto each photomicrograph (cf. Kalman & Keay, 2014). Paxinos and Watson's stereotaxic atlas was used to define the anatomical boundaries of the hippocampus and the dentate gyrus (Paxinos & Watson, 2007). The total volumes of the hippocampus and dentate gyrus were calculated using Cavalieri's method (Rosen & Harry, 1990), in which the surface areas of each of these structures are calculated in equidistant serial coronal sections and then multiplied by the distance between the adjacent sections. The surface areas of the hippocampus on serial coronal sections for its entire rostrocaudal extent are shown in Figure 1c. The total hippocampal volumes calculated from these sections are shown in Figure 1a.

The dorsal hippocampus is defined anteriorly by the presence of CA3 cells at approximately the coronal level represented at −1.72 mm bregma in the atlas of Paxinos and Watson (2007) and posteriorly by the transitional zone of the fimbria and its replacement by the oriens layer of the hippocampus. A distinction aided by the fact that the fibers of the fimbria are parallel to the plane of sectioning and the oriens layer runs perpendicular to the plane of sectioning. The intermediate hippocampus is defined anteriorly by the disappearance of the fimbria and the appearance of the ventral subregions. Its posterior border is defined by the merging of the dorsal and ventral granular layers of the dentate gyrus. This border was selected in order to maintain consistency with the boundaries that we used to subdivide the dentate gyrus into functional subregions. The posterior hippocampus extends from the intermediate hippocampus to its posterior border identified by the disappearance of the molecular layer of the dentate gyrus and the CA1 regions, at approximately −6.8 mm caudal to bregma. The medial borders were defined by the disappearance of the densely labeled pyramidal cells, defining the CA1 region, at approximately −5.16 mm from bregma. In the region of CA1, we defined the border using a perpendicular line drawn from the hippocampal fissure to the alveolus. The lateral border was established by the change in staining between oreins of the hippocampus and the alveolus. At the point where the CA2 region changes into the ventral subiculum a line was drawn between the end of the pyramidal layer and the hippocampal fissure. The boundaries of the dentate gyrus were defined by the border of the molecular and granular layers which are readily identified in thionin‐stained sections. The posterior dentate subregion was defined anteriorly as the point at which the dorsal and ventral regions merge and posteriorly as the point at which the granular layer has disappeared.

Comparisons of sections from randomly selected rats, perfused on different days, were used to compare cortical thickness, the width of the diencephalon, and the distance from the dorsal surface to superior tip of the third ventricle at the same anteroposterior level to ensure that there were no differences in tissue shrinkage between animals. There was no evidence of any variability in the effects of fixation on these tissues.

**Figure 2:**
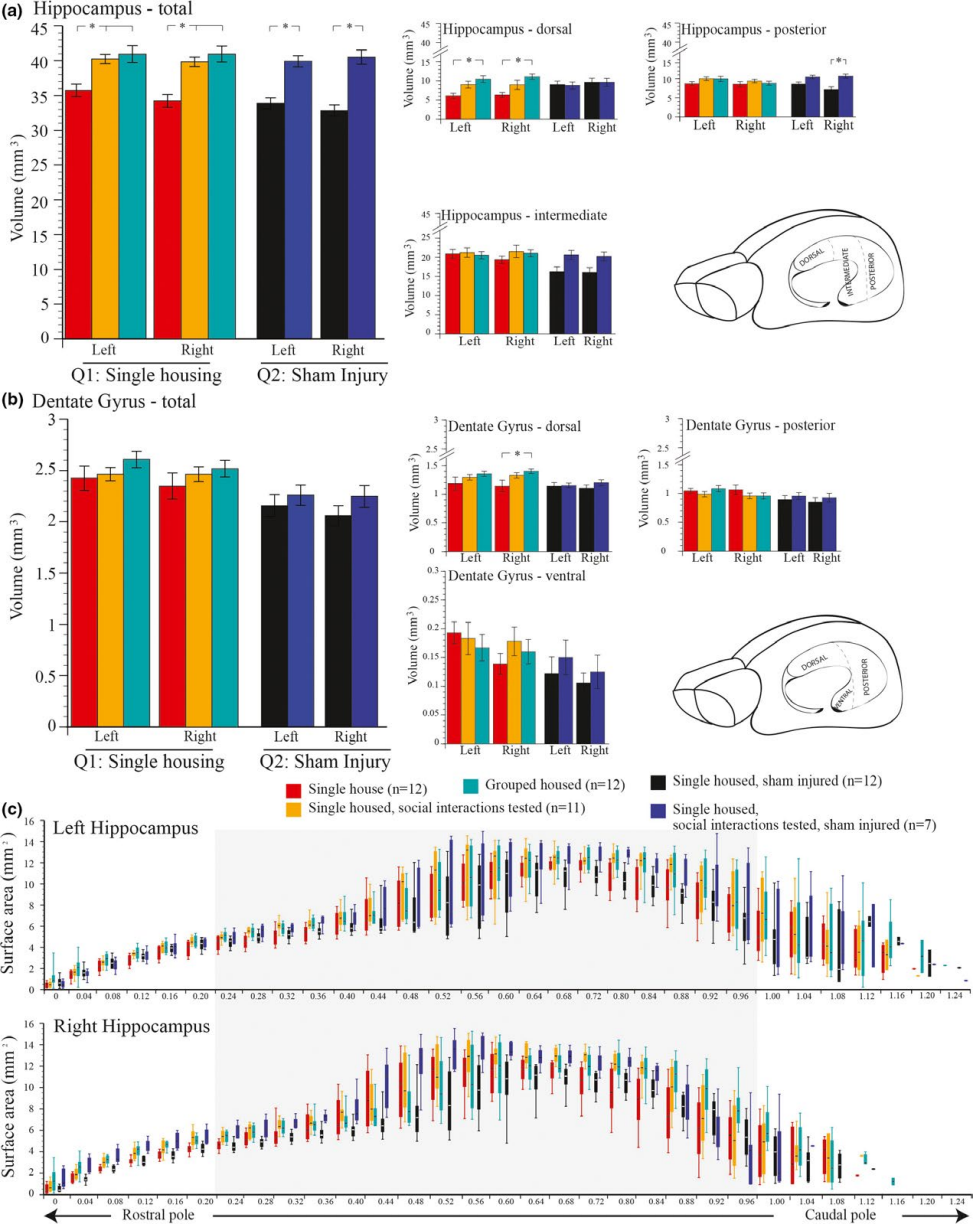
(a, b) Significant differences were detected between experimental groups in the volumes of the dentate gyrus (analysis of variance [ANOVA], left: [*F*_4,49_ = 5.011, *p* < .002], right: [*F*_4,49_ = 5.217, *p* < .001]) and subregion of the dorsal dentate gyrus (ANOVA, left: [*F*_4,49_ = 3.369, *p* < .016], right: [*F*_4,49_ = 5.011, *p* < .002]). Significant differences were also detected in the volumes of the whole hippocampus (ANOVA, left: [*F*_4,49_ = 12.193, *p* < .001], right: [*F*_4,49_ = 17.442, *p* < .001]), dorsal hippocampus (ANOVA, left: [*F*_4,49_ = 3.901, *p* < .008], right: [*F*_4,49_ = 3.821, *p* < .009]), intermediate hippocampus (ANOVA, left: [*F*_4,49_ = 3.341, *p* < .017], right:[*F*_4,49_ = 3.394, *p* < .014]), and the posterior hippocampus (ANOVA, left: [*F*_4,49_ = 2.147, *p* < .089], right: [*F*_4,49_ = 3.492, *p* < .014]). (c) Boxplots showing median value, range, and interquartile range of the surface area (mm^2^) of left and right hippocampal sections from the rostral to caudal poles of rats in groups: (1) single housed (*n* = 12); (2) single housed and social interactions tested (*n* = 11); (3) group housed (*n* = 12); (4) single housed and sham injured (*n* = 12); (5) single housed, sham injured, and social interactions tested (*n* = 7). *p < .05

The volumes of hippocampus and dentate gyrus were compared between all groups using multivariate analysis of variance (MANOVA). This analysis was conducted following preliminary assumption testing for linearity, normality, univariate, and multivariate outliers as well as homogeneity of variance. A MANOVA was performed for the following datasets: (1) left dentate gyrus (dorsal, ventral, posterior), (2) right dentate gyrus (dorsal, ventral, posterior), (3) left hippocampus (dorsal, intermediate, posterior), and (4) right hippocampus (dorsal, intermediate, posterior). Statistical significance was determined for *p* < .05. Where statistical significance was detected within the experimental groups a Tukey's HSD post hoc analysis was used for the subregions of the dentate gyrus and hippocampus. The relationships of hippocampal and dentate gyrus volumes with the expression of dominance, social, nonsocial, and submissive behaviors during the resident–intruder social interactions were also tested. These data were evaluated using Pearson's correlation (where *r* > .44 is considered significant for *N* = 18; Figure 2b).

## 3 RESULTS

Social interactions in the resident–intruder test are stable over the 11‐day testing procedure (see Figure 1a). When exposed to an intruder rat, the resident rats spend almost half of the 6‐min test period engaged in nonsocial behavior, which is predominantly self‐grooming and exploring the cage. Dominance is the next most frequent behavior expressed and comprises mainly of the resident standing on top of the supine intruder, aggressive grooming of the intruder, and chasing and sideways lateral pushing. Negligible time is spent in submissive behaviors (<20 s) and the remainder of the testing period is spent in social behavior, which mainly comprises sniffing around the anogenital region and the face.

Rats singly housed without the opportunity to express the normal repertoire of resident–intruder social behaviors as described earlier had significantly smaller hippocampal volumes than rats that underwent daily resident–intruder testing (Figure 2a). That is, exposure to an intruder in social interactions tested rats was associated with an increased hippocampal volume (Tukey's HSD post hoc testing, left: *p* < .005, right: *p* < .001), which was similar to rats housed in a social group under standard laboratory housing conditions (Tukey's HSD post hoc testing, left: *p* < .001, right: *p* < .0001). We identified that the dorsal hippocampus was significantly smaller in single‐housed (nonbehavioral tested) versus group‐housed rats (Tukey's HSD post hoc testing, left: *p* < .007, right: *p* < .008; see Figure 2a [*Q1*]). With the exception of the right dorsal dentate gyrus (Tukey's HSD post hoc testing, right: *p* < .001), there were no differences in dentate gyrus volumes between single‐ and group‐housed rats (see Figure 2b [*Q1*]).

In sham‐injured rats, the opportunity to express the normal repertoire of resident–intruder social behaviors was associated with an increased total hippocampal volume when compared with sham‐injured rats that had no opportunity to express the normal repertoire of social behaviors in the resident–intruder test (Tukey's HSD post hoc testing, left: *p* < .001, right: *p* < .0001; Figure 2a [*Q2*]). A significant lateralized increase in volume was revealed in the right posterior hippocampus (Tukey's HSD post hoc testing, right: *p* < .006). No significant differences were observed in the volume of the dentate gyrus in any group (Figure 2b [*Q2*]). It is important to note that sham injury alone had no effect on the volume of the hippocampus or dentate gyrus (i.e., compare *Q1* and *Q2* in Figure 2a, b). Furthermore, rats expressing the normal repertoire of resident–intruder social behaviors showed identical hippocampal volumes regardless of having a sham injury or not (see *Q1* vs. *Q2* in Figure 2a, b).

**Figure 3:**
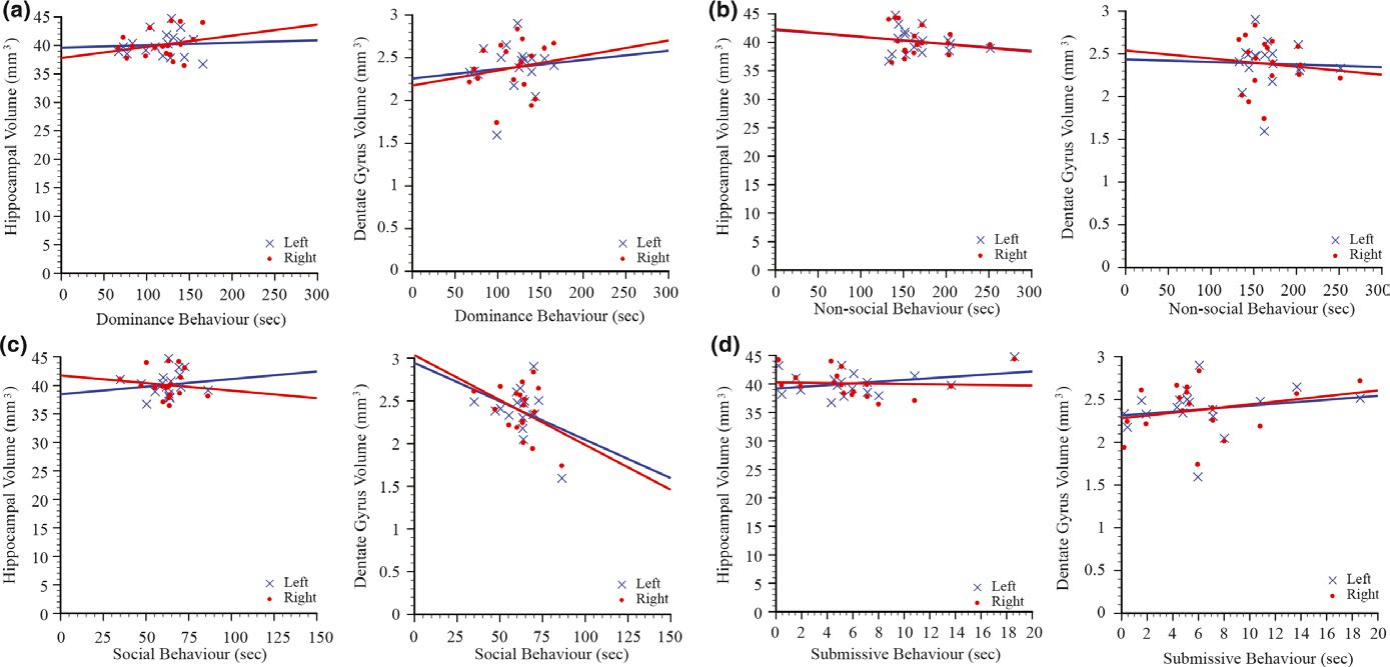
Scatterplots and regression lines demonstrating the relationships between hippocampal or dentate gyrus volumes and time (in seconds) spent in dominance (a), nonsocial (b), social (c), and submissive (d) behaviors in a 6‐min resident–intruder social interaction test

In rats that underwent resident–intruder social interactions testing there was no significant relationship between either the volume of the hippocampus or the volume of the dentate gyrus and the expression of dominance, nonsocial, social, or submissive behaviors for rats that were either singly housed or singly housed and sham injured (Figure 3).

## 4 DISCUSSION

The present data show that single‐housed rats have smaller hippocampi than (1) single‐housed rats that have the opportunity to interact with a conspecific *intruder* in the resident–intruder test and (2) rats housed in a social group under standard laboratory housing conditions. Our earlier data showed reductions in hippocampal volume in nerve‐injured rats with significantly reduced dominance behaviors (Kalman & Keay, 2014). On balance, when taken together, these observations raise the possibility that a component of the interactions with another rat, most likely related to the expression of dominance that conserves hippocampal volume. In fact, when challenged with either nerve injury or surgical (sham) controls, rats that express dominance during social interactions are those that show a preservation of hippocampal volume.

Previous attempts at defining the mechanisms that might be at play in regulating the volume of the hippocampus in the context of behavioral responses have implicated corticosterone levels and altered regulation of the hypothalamic‐pituitary‐adrenal axis. Of note are the reports of reduced hippocampal volume in people with elevated cortisol levels (Tamminga, Nakamura, & Thomas, 2015) associated with depressive disorder and Cushing's disease as well as in animal models of acute and chronic stress that increase corticosterone level (Conrad, 2008). It has been demonstrated in a number of studies that hippocampal volume is regulated by corticosteroid‐dependent mechanisms (Brown, Rush, & McEwen, 1999; Brown et al., 2015; Zhang, Zhao, & Wang, 2015). In these reports, corticosteroid levels have been experimentally increased or decreased from the usual ranges associated with normal behavioral and physiological activity. The consequent changes in hippocampal volume have been explained in terms of altered neuronal proliferation and survival, altered expression of cytoskeletal proteins, dendritic branching and numbers of synapses, endothelial cell proliferation and angiogenesis, and on astrocyte numbers (Czéh & Lucassen, 2007; Sapolsky, 2000). We assume, as other have, that our observations of changes in volume reflect combinations of altered structure and activity of the underlying neuronal, astrocyte, microglial, and vascular cellular components, (which may include the processes of neurogenesis) and possibly altered intra‐ and extracellular fluid content.

In animal models the impact of acute and chronic stressors can be modified by interactions with a conspecific, this has been termed social buffering. This effect may also be observed in people, in so far as social support networks are known to buffer the impact of stressors on individuals. It has been suggested recently that the corticosterone response to stress can be attenuated by social interactions via an oxytocin‐dependent mechanism (DeVries, Glasper, & Detillion, 2003; Neumann, 2002; Timmer, Cordero, Sevelinges, & Sandi, 2011). The emerging link between social buffering, oxytocin and corticosterone, may contribute to the hippocampal volume changes that are described in this study. Sánchez‐Vidaña et al. (2016) have demonstrated that elevating corticosterone in Sprague Dawley rats suppressed cell proliferation in the hippocampus and decreased social interactions in response to a conspecific in a neutral environment. The social interactions described by Sánchez‐Vidaña et al. (2016) include behaviors that we have categorized as dominance and social behaviors in the present study. They also showed that oxytocin administration in rats (1) reversed the suppression of cell proliferation in the hippocampus and (2) doubled the frequency of social interactions with a conspecific (i.e., social and dominance behaviors) in a neutral environment when compared to the number of interactions observed in nontreated controls. Furthermore, administration of oxytocin was shown to increase the number of dendritic branches on immature developing hippocampal neurons, which has been suggested to result in increased hippocampal volume. In further support of a role for oxytocin in dominance behavior, male mice in which the oxytocin gene has been knocked out have been shown to display lower levels of dominance behavior and an increased expression of nonsocial behaviors in the resident–intruder social interactions test (Lazzari et al., 2013).

Thus, the impact of corticosteroids on hippocampal volume and their underlying etiologies can be modulated by oxytocin, whose expression levels are related in turn to the expression of the full repertoire of resident–intruder social interactions. Whether the relationships between behavioral expression and hippocampal volume described in our data can be explained by these relationships is an important question. Determining the exact directionality of these inter‐relationships for the data we present would be a critical next step beginning with the measurement of oxytocin levels in our experimental groups.

The critical question is the nature of the relationship between the complete and ethologically appropriate expression of the behavioral repertoire and the preservation of hippocampal volume. It is clear that exposure to a conspecific alone is not sufficient to maintain hippocampal volume in the protocols described and that some element of the expression of the social behavioral repertoire is linked to hippocampal volume. The relationship between hippocampus, social behavior, behavioral sequencing, and appropriate response selection is well recognized, however, our data suggest that the neural circuitry of the hippocampus may not only drive these functions, but that their integrity is responsive to the physical expression of such outputs.

Our data serve to highlight the need for critical evaluation of each procedural element of behavioral experimental protocols, as they may unexpectedly influence experimental outcomes.

## CONFLICT OF INTEREST

The authors declare they have no conflicts of interest.
